# Porous MOF Microneedle Array Patch with Photothermal Responsive Nitric Oxide Delivery for Wound Healing

**DOI:** 10.1002/advs.202103449

**Published:** 2021-11-16

**Authors:** Shun Yao, Yuetong Wang, Junjie Chi, Yunru Yu, Yuanjin Zhao, Yuan Luo, Yongan Wang

**Affiliations:** ^1^ State Key Laboratory of Toxicology and Medical Countermeasures Beijing Institute of Pharmacology and Toxicology Beijing 100850 China; ^2^ Department of Rheumatology and Immunology Institute of Translational Medicine The Affiliated Drum Tower Hospital of Nanjing University Medical School Nanjing 210008 China; ^3^ State Key Laboratory of Bioelectronics School of Biological Science and Medical Engineering Southeast University Nanjing 210096 China; ^4^ Wenzhou Institute University of Chinese Academy of Sciences Wenzhou Zhejiang 325001 China

**Keywords:** graphene oxide, HKUST‐1, microneedles, nitric oxide, wound healing

## Abstract

Patches with the capacity of controllable delivering active molecules toward the wound bed to promote wound healing are expectant all along. Herein, a novel porous metal‐organic framework (MOF) microneedle (MN) patch enabling photothermal‐responsive nitric oxide (NO) delivery for promoting diabetic wound healing is presented. As the NO‐loadable copper‐benzene‐1,3,5‐tricarboxylate (HKUST‐1) MOF is encapsulated with graphene oxide (GO), the resultant NO@HKUST‐1@GO microparticles (NHGs) are imparted with the feature of near‐infrared ray (NIR) photothermal response, which facilitate the controlled release of NO molecules. When these NHGs are embedded in a porous PEGDA‐MN, the porous structure, larger specific surface area, and sufficient mechanical strength of the integrated MN could promote a more accurate and deeper delivery of NO molecules into the wound site. By applying the resultant NHG‐MN to the wound of a type I diabetic rat model, the authors demonstrate that it is capable of accelerating vascularization, tissue regeneration, and collagen deposition, indicating its bright prospect applied in wound healing and other therapeutic scenarios.

## Graphical abstract

A porous metal‐organic framework (MOF) microneedle (MN) patch is presented for wound healing. Due to the photothermal response of NO@HKUST‐1@GO microparticles (NHGs), the NHGs embedded MN patch could promote a more accurate delivery of NO molecules into the wound site through near‐infrared ray (NIR) irradiation. Thus, such MOF MN patch offers a promising approach in diabetic wound healing.

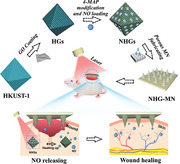

## 1 Introduction

Hard‐to‐healing cutaneous wounds, especially chronic diabetic wounds, often bring on increasing family and social burden worldwide. Thus, the wound remodeling and regeneration course have attracted great attention in the biomedical and clinical fields.^[^ ^1^ ^]^ During the general healing process, numerous promoting factors or mechanisms have been employed for tissue restoration, such as endothelial cell growth factor (VEGF), platelet‐derived growth factor (PDGF), nitric oxide (NO), oxygen (O_2_), and so on.^[^ ^2^ ^]^ Among them, NO, usually involving in the comprehensive physiological and pathological process as an endogenous gaseous molecule, possesses multi‐functions including vasodilation and angiogenesis, signal transmission and integration, infection elimination, and immunoregulation, etc.^[^ ^3^ ^]^ As these characteristics endow NO with dramatic value in the wound healing process, various NO‐loading platforms (e.g., liposomes, silica particles, dendrimers, hydrogel films, inverse opal scaffolds, and microneedle arrays) have been developed to execute precise control over the NO delivery.^[^ ^4^ ^]^ Although with many achievements, it remains a challenge to obtain the controlled release and deeper

diffusion of NO on the wound site due to its short half‐life and plain functions of those platforms. Thus, multifunctional platforms with controllable NO delivery are still anticipated in the wound healing field.^[^ ^5^ ^]^

**Figure 1:**
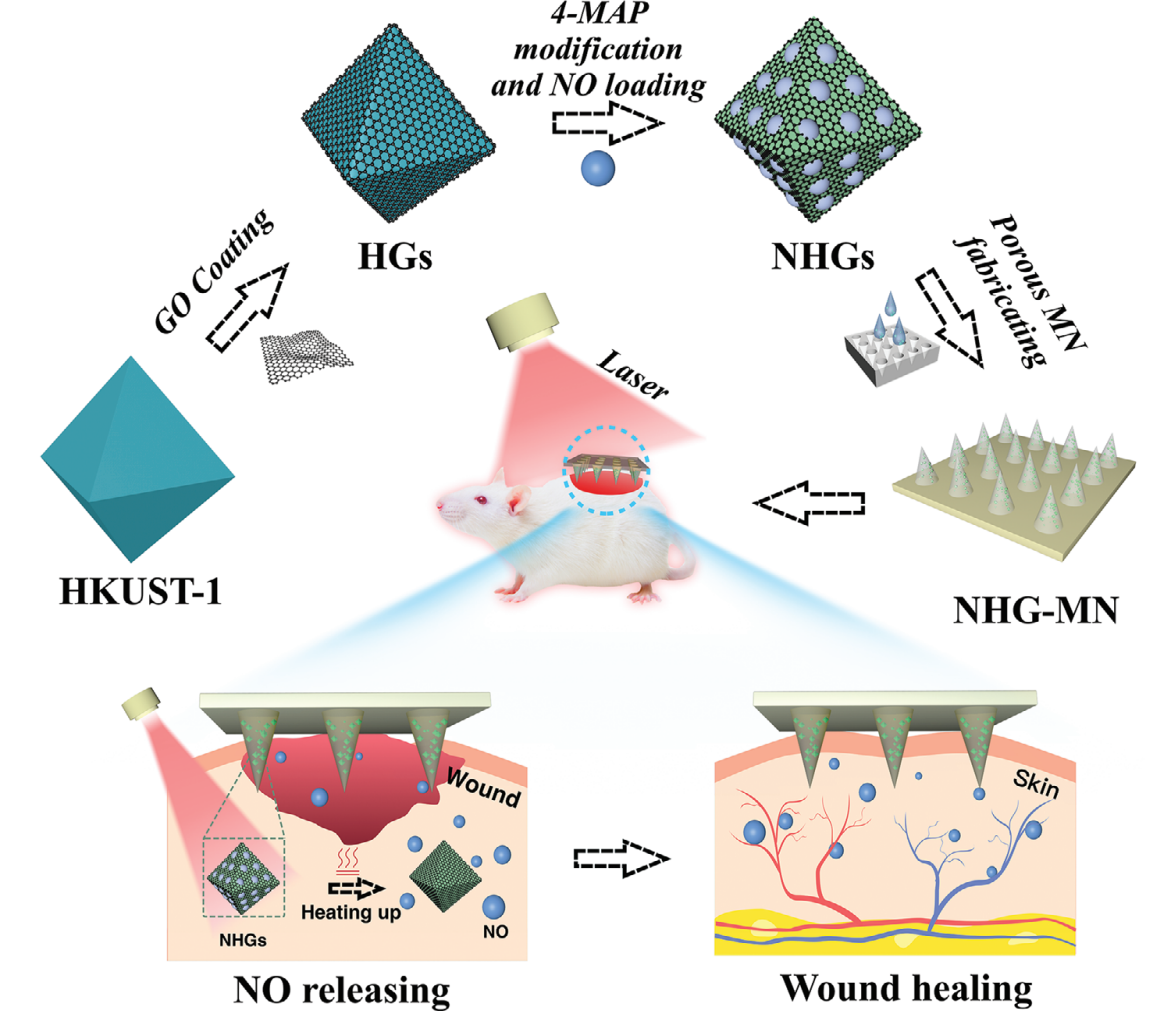
Schematic diagram of the preparation and application of the porous MOF MN array, which was fabricated by PEGDA and encapsulated with NHGs via a template infusion method.

In this paper, we proposed a novel porous metal‐organic framework (MOF) microneedle array (MN) patch enabling photothermal‐responsive NO delivery for wound healing, schemed in **Figure** **1**. MN is an emerging transdermal drug delivery system, characterized by micron‐level needle‐like protrusions densely ranking into an array.^[^ ^6^ ^]^ Particularly, porous MN manufactured from pore‐forming materials comprises large numbers of pores or gaps inside the needle‐tips and base, facilitating the loading of active substance. As a composition of metal nodes and organic ligands, MOF possesses multifarious outstanding features, such as ultrahigh porosity, large specific surface area, and high thermal conductivity. Benefiting from these superiorities, MOF has been extensively employed in hydrogen storage, wastewater treatment, catalytic reaction, and so on.^[^ ^7^ ^]^ Especially, several kinds of MOFs, such as copper‐benzene‐1,3,5‐tricarboxylate (HKUST‐1), could preserve the capacity of NO adsorption and separation.^[^ ^8^ ^]^ However, these MOFs usually failed in the controlled NO discharge. Thus, seeking an approach to solve the problem of MOFs for controllable gas release, and integrating them into the MN patch, would hopefully exploit a novel intelligent NO release system for the treatment of refractory wounds.^[^ ^9^ ^]^

Herein, we employed a responsive graphene oxide (GO) to encapsulate HKUST‐1, obtained the NO@HKUST‐1@GO microparticles (NHGs), and loaded them in a typical poly (ethylene glycol) diacrylate (PEGDA)‐based porous MN patch to realize the near‐infrared ray (NIR)‐controlled NO delivery (Figure 1). As a favorable photothermal responsive material, the GO shell imparts the NHGs with the property of temperature modulating under NIR, thereby facilitating the controlled release of NO molecules. By using ultraviolet (UV) curing, these NHGs could be encapsulated in PEGDA‐MN with no influence on their NO release ability. The porous structure, larger specific surface area, and sufficient mechanical strength of the integrated MN could promote a more accurate and deeper delivery of NO molecules into the wound site. When applying the resultant MN to diabetic wounds, it was found that the wound closure rate in the porous NHG‐MN+NIR group could reach 99% within 13 days. In addition, histological analysis exhibited their outstanding performance in inflammation elimination, neovascularization, and collagen deposition. These motivative results indicated that the porous NHG‐MN patch integrated with photothermal responsive GO possessed great superiorities in controlled NO release, offering a promising approach in diabetic wound healing and other clinical applications.

## 2 Results and Discussion

**Figure 2:**
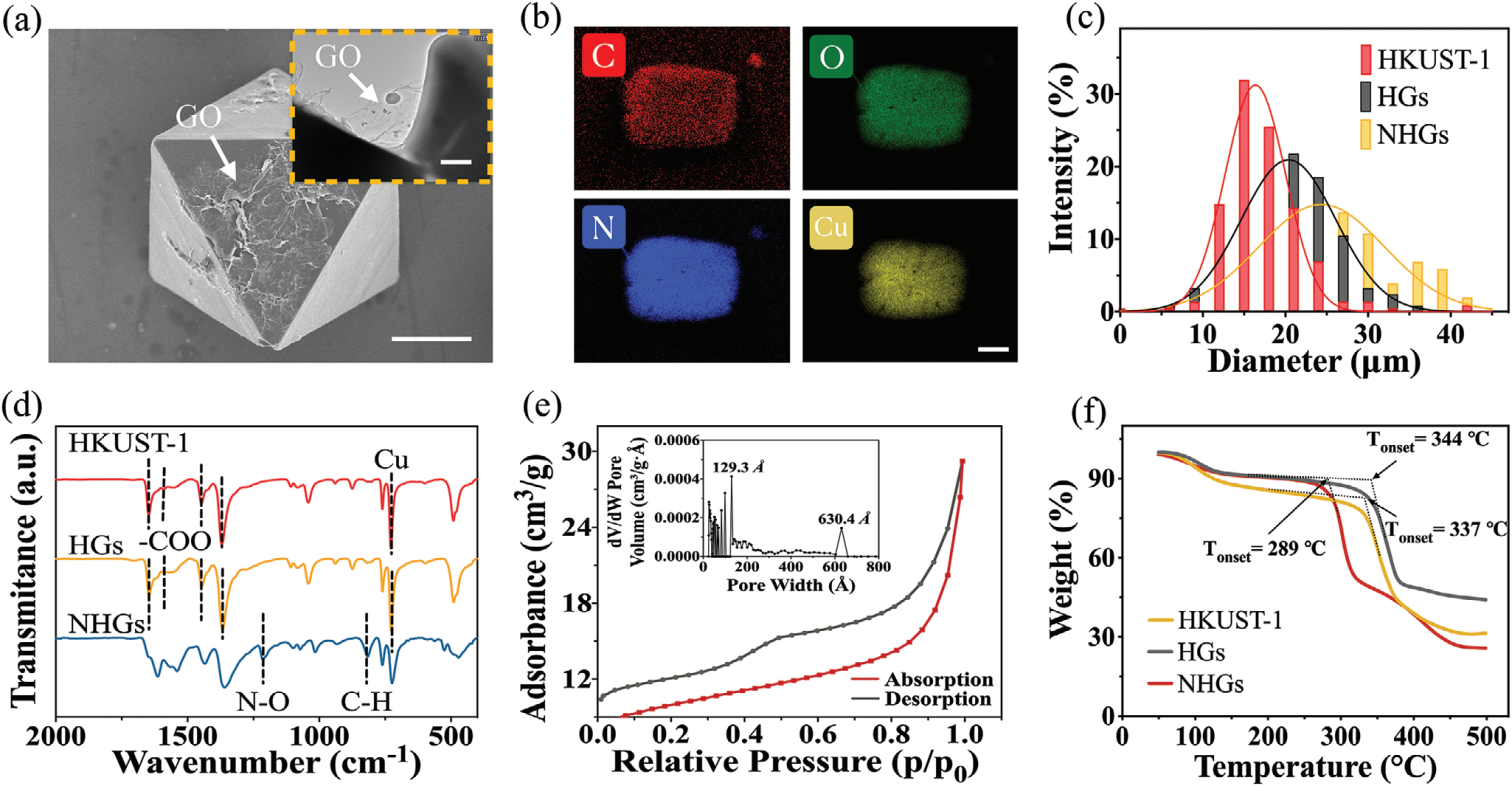
Synthesis and functional characterization of the HKUST‐1‐derived microparticles: a) SEM image of individual HGs (Inset: TEM image of HGs surface); b) EDS analysis of HGs; c) The size distribution of the HKUST‐1‐derived microparticles; d) FTIR spectra of the HKUST‐1‐derived microparticles; e) The nitrogen absorption curve and porosity of NHGs; f) Thermogravimetric analysis of the HKUST‐1‐derived microparticles. Scale bars are 10 µm in (a), 1 µm in the inset image, and 2 µm in (b).

In a typical experiment, the copper nitrate trihydrate (Cu(NO_3_)_2_), trimesic acid (BTC), GO, and 4‐pyridinemethaneamine (4‐MAP) were used as raw materials to synthesize the NO carrier through multi‐step hydrothermal and high‐pressure reaction (Figure 1). The SEM images in Figure S1c (Supporting Information) revealed that the HKUST‐1 microparticles displayed a typical octahedral shape with smooth facets and sharp edges. When the single‐layer GO suspension was added to the synthetic system, it could be observed that the surface of HKUST‐1 crystal was covered by a thin layer of GO film, exhibiting an inhomogeneous folded structure (**Figure** **2**a). As shown in Figure 2b, the existence of C, O, N, and Cu elements possessed by the resultant HKUST‐1@GO microparticles (HGs) was further verified based on the evidence of Energy‐dispersive X‐ray spectroscopy (EDS). The HGs were modified with 4‐MAP, placed in a NO environment, forming the resultant NHGs. During the synthesis and modification process of NHGs, the color of the HKUST‐1‐derived microparticles changed significantly. The introduction of GO converted the color of HKUST‐1 dispersion from light blue to black. Subsequently, due to the ligand exchange reaction of 4‐MAP, the color turned green, with no obvious change on the microstructure of GO layer (Figure S1a–c, Supporting Information). The diameter of HKUST‐1‐derived microparticles gradually increased from 17.5 to 27.7 µm due to the post‐modification of GO and 4‐MAP, but the structure of the regular octahedron remained unchanged (Figure 2c and Figure S1c, Supporting Information). These results proved that the post‐modification did not destroy the primary structure of HKUST‐1 base.

The combination of high specific surface area and adjustable aperture endows MOF with great advantage as a storage and transport system for NO. After the surface modification of 4‐MAP, NO molecules will be adsorbed by forming *N*‐diazeniumdiolates (NONOates) groups (Figure S2a, Supporting Information). Stimulated by water, light or heat, NONOates can release two equivalents NO molecules under physiological conditions, thus enabling controlled delivery of NO. To further confirm the successful loading of NO, a Fourier Transform Infrared Spectroscopy (FTIR) analysis of HKUST‐1‐derived microparticles was conducted (Figure 2d). The spectroscopy graph of HKUST‐1 showed the characteristic absorption peaks at 1365, 1434, 1530, 1650 cm^−1^, which corresponded to the vibrations of carboxylic acid groups in BTC. As the tiny addition of GO, its characteristic peak did not be detected, which rendered the FTIR spectra of HGs and HKUST‐1 very similar. After the modification of 4‐MAP and the loading of NO, additional transmission peaks appeared at 763 and 1202 cm^−1^, representing the C—H bond in 4‐MAP and the N—O bond in NONOates, respectively. Additionally, X‐ray diffraction (XRD) spectrum was used to confirm the crystal structure of HKUST‐1‐derived microparticles. Results showed that the characteristic diffraction peak appeared at 2*θ* of 5˚–30˚, which was consistent with the typical structure of HKUST‐1 crystal. Moreover, after GO modification and NO loading, the position of the diffraction peak was retained, indicating the crystal structure had not been destroyed (Figure S2b, Supporting Information). The FTIR and XRD results showed that 4‐MAP modified HGs successfully trapped NO molecules by forming NONOates groups.

The nitrogen adsorption experiment before and after NO loading was also measured to explore the specific surface area and porosity of HKUST‐1‐derived microparticles. As revealed in Figure S2c (Supporting Information), the nitrogen adsorption curve of HKUST‐1 increased rapidly before NO loading, and reached a plateau instantly at a relatively lower pressure region, representing the classical type‐I adsorption isotherms with an average micropore size of 28.7 Å. However, the nitrogen adsorption curve of NHGs exhibited mesoporous characteristics with an average pore size of 107.6 Å (Figure 2e). This could be explained by that, the modification of 4‐MAP and loading of NO blocked the micropores, leaving the mesoporous structure on the surface. The thermal stability of HKUST‐1‐derived microparticles was measured by thermogravimetric analysis (TGA). As demonstrated in Figure 2f, owing to the removal of bonding water, the particle mass reduced by 10% within 100 °C and the onset decomposition temperature (*T*_onset_) of HKUST‐1, HGs, and NHGs were 337, 344, and 289 °C, respectively. The outstanding thermal stability enabled them to maintain structural integrity during the high‐temperature synthesis process and in harsh application scenarios, thereby ultimately ensuring their excellent NO adsorption performance.

**Figure 3:**
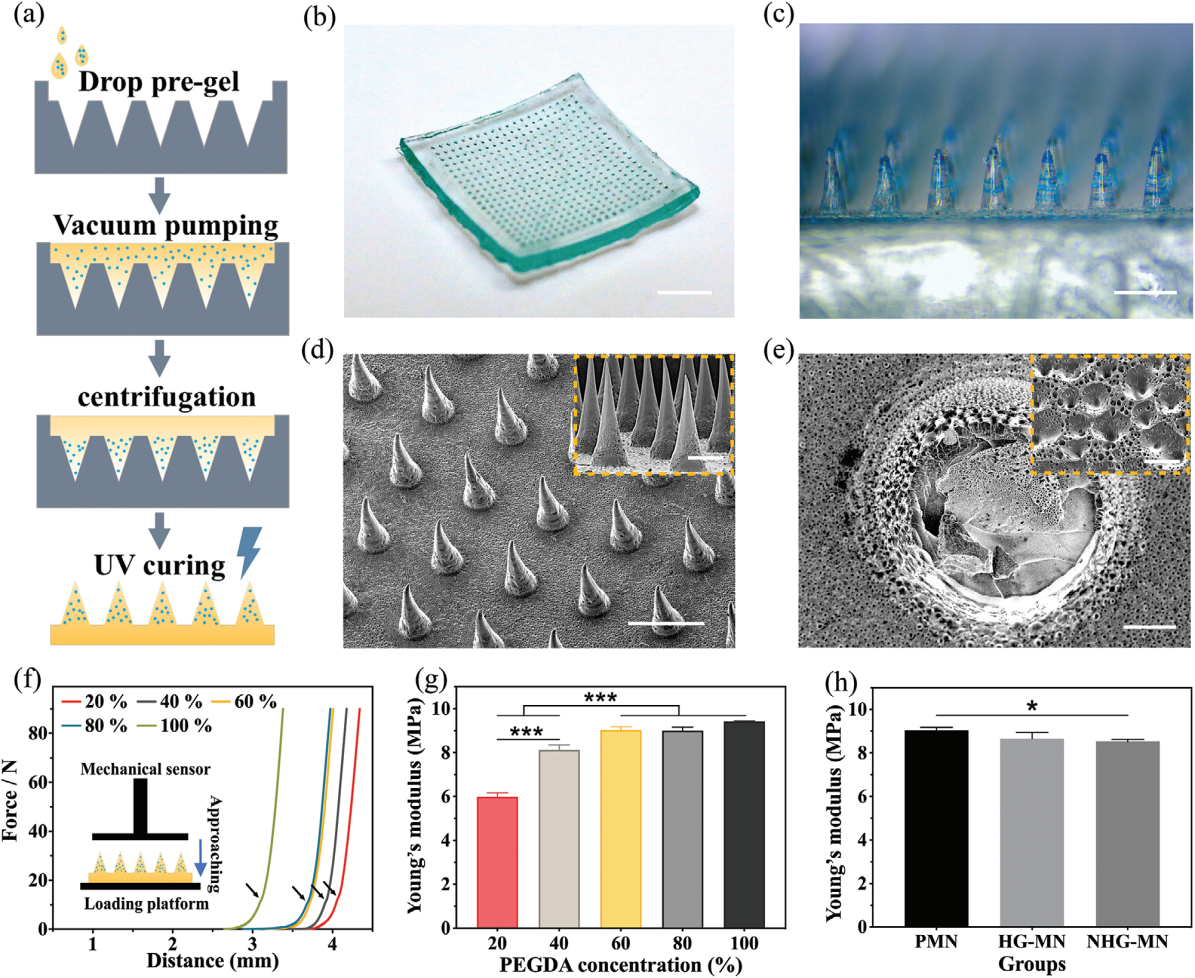
Characterization of the porous MN patch: a) Schematic illustration of the preparation process of the NHG‐MN patch; Optical photography of b) the NHG‐MN, and c) its amplified needle‐tips; d) SEM images of the porous MN patch; e) SEM images revealed the porous channels distributed on the porous MN patch; f) Force‐displacement curves of MN with different PEGDA concentrations (Inset: Schematic diagram of the mechanical strength test). The arrows indicated the respective tiny plateau; g) Young's modulus of MN patch with gradient PEGDA concentrations; h) Young's modulus of PMN, HG‐MN, and NHG‐MN patches. Data are shown as mean ± SD (*n* = 3) and analyzed using the one‐way ANOVA test. Significances are presented by *p < 0.05, **p < 0.01, ***p < 0.001. Scale bars are 5 mm in (b), 500 µm in (c), 500 µm in (d) (200 µm in inset), 50 µm in (e) (15 µm in inset).

To ensure the accurate delivery of NO molecules to the wound bed, we encapsulated NHGs inside a porous PEGDA‐MN patch. In detail, NHGs were blended with PEGDA solution by ultrasound, and uniformly distributed in the precursor. Then, ammonium bicarbonate and glacial acetic acid were added to create massive microbubbles, which were utilized to generate the porous structure of MN. After the needle tips were infilled with the pre‐gel solution by a vacuum pump, the NHGs were deposited into the needle tips via low‐speed centrifugation. Finally, the porous MN patch encapsulated NHGs was prepared by UV solidification (**Figure** **3**a). In the optical images, the needle tips were arranged neatly in an array of 15 × 15 mm, with NHGs distributed within them, as shown in Figure 3b,c. Scanning electron microscope (SEM) images further verified the obvious porous structure located on the MN surface (Figure 3d,e). These holes were conducive to NO release because they facilitated the ultrahigh surface area and unblocked gas channel when MN interacted with the wound bed. It was verified that the porous MN patch made from 60% PEGDA pre‐gel exhibited the most abundant pores in the case of the same proportion of porogen, which could be explained by that 60% PEGDA pre‐gel provided the most suitable viscosity for the generation of tiny bubbles (Figure S3a,b, Supporting Information).

The holes distributing on the tips may compromise the mechanical property of the porous MN patch and thus affect the transdermal puncture ability. To evaluate the mechanical property of porous MN patches under the condition of the same porogen content, the maximum strain capacity of their needle tips was tested by an electronic tension testing machine. During the measurement, the porous MN patch was placed on a horizontal table with needle tips pointed toward the pressure sensor which gradually descended. As soon as the two touched, the pressure data within 500 µm would be recorded (Figure 3f). The mechanical strength of porous MN patches with different PEGDA concentrations was tested by measuring their force‐displacement curves and calculating the respective Young's modulus. As shown in Figure 3f, there was a tiny plateau at the midpoint of the force‐displacement curve, indicating that a small breakage appeared on the needle tips while the whole body could still resist the total force during compression. A higher concentration of PEGDA‐MN exhibited larger Young's modulus, which was proved more suitable for the manufacture of porous MN patches that required higher mechanical strength (Figure 3g). In addition, porous MN encapsulated with NHGs could hardly change their mechanical strength, as shown in Figure 3h. When the porous MN patches inserting into the simulated skin, there exhibited neatly arranged grooves (Figure S4a, Supporting Information). The laser scanning confocal microscopy (LSCM) results also confirmed that the depth of MN penetrating the skin reached 240 µm, which ensured adequate depth for NO delivery (Figure S4b–f, Supporting Information). Besides, the transportation of NO at the wound bed required water as the medium, thus it is requisite to study the hydrophilicity of the porous MN surface. As shown in Figure S3c (Supporting Information), the maximum water contact angle did not exceed 45°, indicating excellent hydrophilicity in all groups. Therefore, the PEGDA pre‐gel with a concentration of 60% was chosen as the optimized condition to fabricate the porous MN patch.

**Figure 4:**
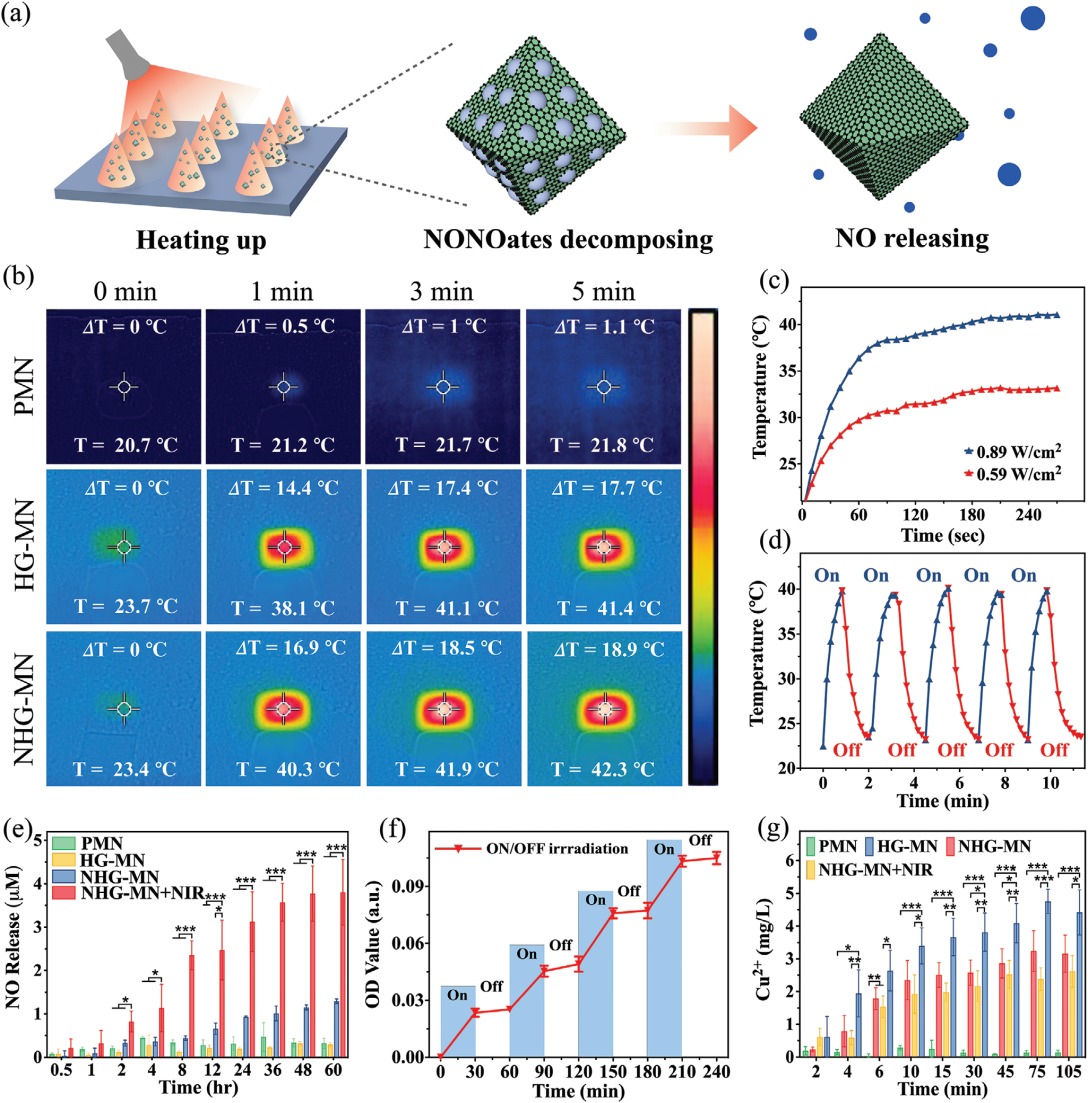
Photothermal‐responsive NO delivery of porous MOF MN patches: a) Schematic diagram of photothermal‐responsive NO delivery process; b) Infrared thermal photos of MN patch stimulated by NIR irradiation; c) Photothermal responsive profiles of the NHG‐MN; d) Temperature variation of NHG‐MN during 5 photothermal cycles. e) The cumulative NO release amount in different groups (PMN, HG‐MN, NHG‐MN, NHG‐MN+NIR). f) NO release condition of NHG‐MN under intermittent NIR irradiation. g) The cumulative Cu^2+^ release amount in different groups (PMN, HG‐MN, NHG‐MN, NHG‐MN+NIR). Data are shown as mean ± SD (*n* = 3) and analyzed using the one‐way ANOVA test. Significances are presented by *p < 0.05, **p < 0.01, ***p < 0.001.

The GO‐coated NHGs endowed the MN carrier with excellent photothermal ability under NIR exposure and facilitated the controlled release of NO (**Figure** **4**a). To study the temperature‐responsive capacity of MN, we took infrared thermal photos at different time points for temperature monitoring (Figure 4b). After 5 mins’ NIR irradiation with a power density of 0.89 W cm^−2^, the temperature of pure PEGDA‐MN patch (designated as PMN) rose by only 1.1 °C, showing a poor photothermal response. When MN patches containing 1 mg mL^−1^ HGs or NHGs (designated as HG‐MN and NHG‐MN) were irradiated for 5 min at the same power density, their temperature rapidly reached over 40 °C and then stabilized. According to the results of the photothermal curve (Figure 4c), the temperature upper limit of the NHG‐MN increased with the elevated NIR power density gradient, indicating a facile way of tuning temperature via irradiation. It was noteworthy that when the power density was maintained at 0.89 W cm^−2^, the temperature of the MN patch would not exceed 42 °C, which helped to protect the wound from being scalded. Several continuous on/off cycles also confirmed that the NHG‐MN was able to be thermal‐activated repeatedly and steadily by NIR irradiation (Figure 4d). Therefore, the power density of 0.89 W cm^−2^ was selected as a suitable condition for the followed‐up experiments.

To investigate the NO release profile in different MN groups (PMN group, HG‐MN group, NHG‐MN group, and NHG‐MN+NIR group), MN were stored in a sealed environment protected by nitrogen (N_2_) and the real‐time release of NO was calculated at specific time intervals by measuring the dissolving nitrite converted from NO. A nitrite standard curve was established through the Griess assay (Figure S5a, Supporting Information). It was noticed that the cumulative release of NO in the NHG‐MN group and NHG‐MN+NIR group was markedly higher, indicating a superior NO loading capacity of NHG‐MN (Figure 4e). Due to the photothermal effect of GO, the high temperature greatly stimulated the dissociation of NO molecules from 4‐MAP, hence the cumulative release of NO in the NHG‐MN + NIR group was about three times as large as the NHG‐MN group. NO release kinetics under intermittent NIR irradiation was also investigated. Results showed that the NO‐releasing rate possessed a remarkable on/off switching effect, elucidating the NIR irradiation was the trigger to modulate the release of NO (Figure 4f).

Free copper ions (Cu^2+^) can be decomposed from HKUST‐1‐derived microparticles and influence the wound healing process. The release of Cu^2+^ was determined by bis(cyclohexanone) oxalyldihydrazone (BCO) spectrophotometric method. Through the conversion of standard curve (Figure S5b, Supporting Information), the Cu^2+^ in all groups (PMN group, HG‐MN group, NHG‐MN group, and NHG‐MN+NIR group) was completely released within an hour according to the releasing profile. Due to the lack of 4‐MAP modification, the HG‐MN group exhibited the most cumulative Cu^2+^ release amount (Figure 4g). Although extremely low concentrations of Cu^2+^ were beneficial to the proliferation of keratinocytes, high concentrations of Cu^2+^ have a toxic effect on cell growth. The MN, encapsulated with HKUST‐1‐derived microparticles, was then soaked in the Dulbecco's modified Eagle's medium (DMEM), and their biocompatibility was tested by a 3‐(4,5‐dimethylthiazol‐2‐yl)‐2,5‐diphenyltetrazolium bromide (MTT) assay. Because of the toxic effects from high concentrations of Cu^2+^, the OD value and cell viability in the HG‐MN group were significantly decreased after that the materials were co‐cultured with NIH/3T3 cells within 3 days (Figure S6a,b, Supporting Information). Due to the modification of 4‐MAP, the release amount of Cu^2+^ fell sharply, which explained the higher cell viability in the NHG‐MN group and NHG‐MN+NIR group. These results fully demonstrated the excellent operability and favorable biocompatibility of the porous NHG‐MN patch, which can be utilized as a promising photothermal‐responsive NO carrier.

**Figure 5:**
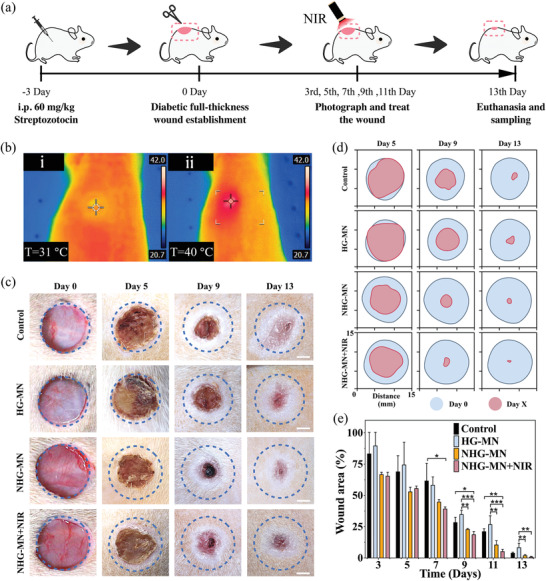
Evaluation of the porous MOF MN patch on wound healing: a) Schematic diagram of in vivo experiment; b) In vivo photothermal images of MN patch before (i) and after (ii) 5 min NIR irradiation (0.89 W cm^−2^); c) Optical images recorded during wound healing process on zero, 5th, 9th, and 13th day; d) Simulation of wound morphological changes within 13 days; e) The wound closure rate among different groups within 13 days. Scale bars are 3 mm in (c). Data are shown as mean ± SD (*n* = 3) and analyzed using the one‐way ANOVA test. Significances are presented by *p < 0.05, **p < 0.01, ***p < 0.001.

In vivo experiments were conducted to verify the practical value of the porous MOF MN patch. As shown in **Figure** **5**a, we established a type I diabetic rat model and excised rat dorsal skin into four parallel circular wounds with a diameter of 1 cm by the scalpel. Then, different groups of rats were randomly assigned: control, HG‐MN, NHG‐MN, NHG‐MN+NIR, and the role of PMN in wound healing was also explored (Figure S7, Supporting Information). The working temperature of the MN patch can be stabilized at about 40 °C under the NIR irradiation with the power density maintaining at 0.89 W cm^−2^ (Figure 5b). In the meantime, the healing condition was recorded by the wound closure rate within the 1st to 13th day at constant intervals. Judging from the dynamic changes of wound morphology, the healing rate in the NHG‐MN group and NHG‐MN+NIR group was faster than in other groups (Figure 5c,d). Quantitative analysis informed that the relative wound area in control, HG‐MN, NHG‐MN, and NHG‐MN+NIR groups were reduced to 4.0 ± 0.6%, 8.5 ± 3.3%, 2.1 ± 0.5%, and 1.0 ± 0.3% during the first 13 days, respectively (Figure 5e). The results suggested that the NHG‐MN group and the NHG‐MN+NIR group, which loaded NO molecules, were more favorable for diabetic wound healing. As photothermal stimulation can release NO more efficiently and controllably, the NHG‐MN+NIR group exhibited the best wound healing effect within all groups. It was not only due to the multifunction of NO in promoting angiogenesis and vasodilation, but also the precise NO delivery ability possessed by the porous MN patch. Meanwhile, owing to the Cu^2+^ toxicity caused by the decomposition of HKUST‐1, the wound healing rate in the HG‐MN group was significantly lower than the control group, which corresponded to the phenomenon of the biocompatibility test.

**Figure 6:**
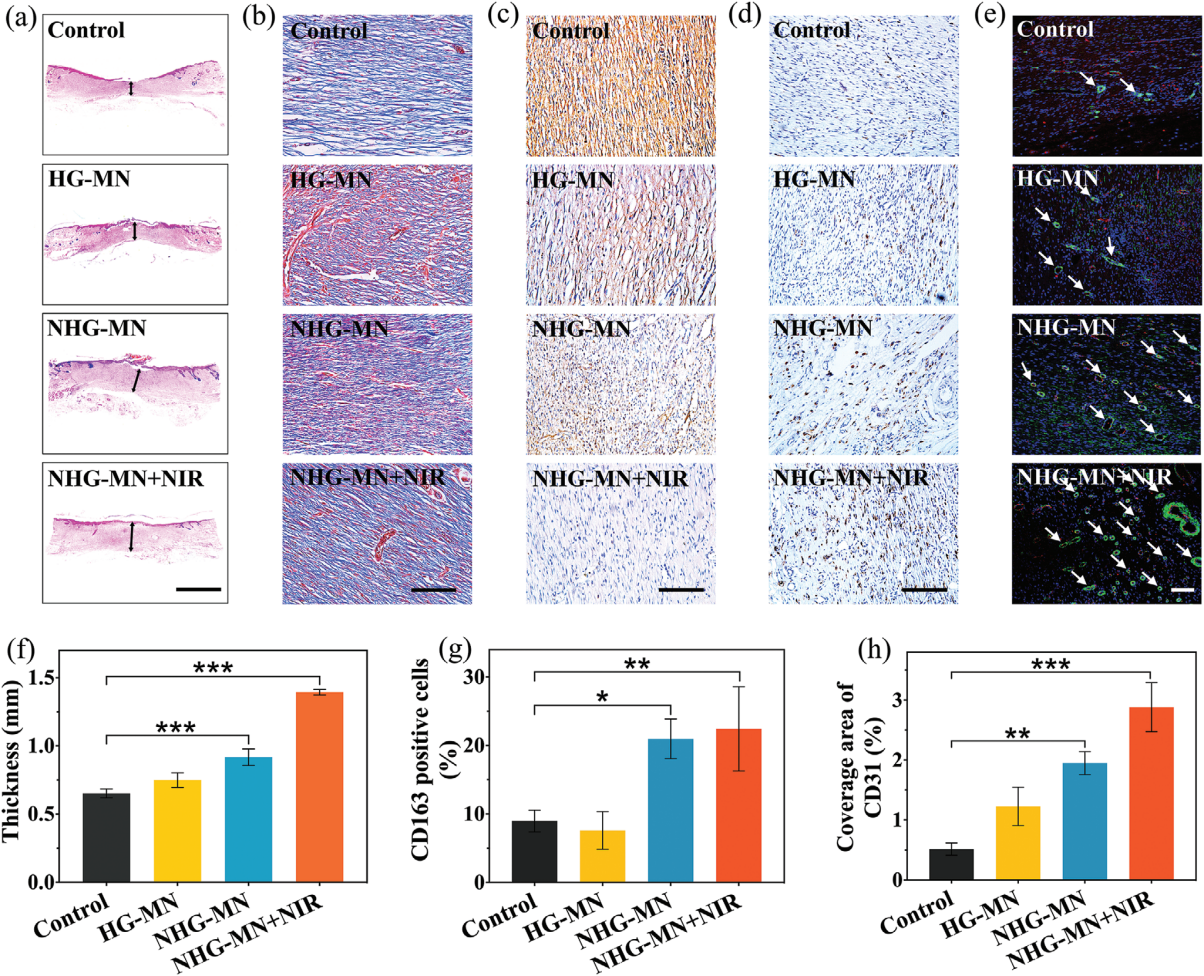
Biological mechanism research on the wound repairment: a) H&E staining of healed skin in different groups; b) Masson trichrome staining in different groups; c) Immunohistochemical staining of IL‐6 secretion for the analysis of inflammation; d) Immunohistochemical staining of CD163 for the analysis of M2 macrophages; e) Immunofluorescent staining of CD31 (red), and *α*‐SMA (green) for the analysis of vascularization; f) Quantitative analysis of epithelial tissue thickness; g) Quantification analysis of CD163 positive cells; h) Quantitative evaluation of the coverage area of CD31. Scale bars are 2 mm in (a), and 100 µm in (b)–(e). Data are shown as mean ± SD (*n* = 3) and analyzed using the one‐way ANOVA test. Significances are presented by *p < 0.05, **p < 0.01, ***p < 0.001.

A key step of skin wound healing is the migration of keratinocyte from surrounding tissue to the wound site, facilitating re‐epithelialization. As an endogenous gasotransmitter, NO molecules play a central role in inflammatory regulation to recruit keratinocyte in the downstream. The reduced function of keratinocytes caused by NO deficiency is an important factor interfering with the healing rate of diabetic wounds. Hematoxylin‐eosin (H&E) staining was used to analyze the performance of re‐epithelization and granulation tissue formation. As shown in **Figure** **6**a, the new stratum corneum in the NO treatment groups (NHG‐MN and NHG‐MN+NIR group) was the most complete and the thickness of granulation tissue was also the largest compared with other groups. Quantitative analysis showed that the healing condition in the NHG‐MN+NIR group was the best, whose granulation tissue thickness reached 1.39 ± 0.02 mm (Figure 6f), indicating that the NIR irradiation accelerated the wound restoration process by promoting the release of NO. The deficiency of inducible nitric oxide synthase (iNOS) in diabetic wounds often leads to inhibition of collagen synthesis and extracellular matrix formation. Masson's trichrome staining (MTC) in the NHG‐MN+NIR group demonstrated that a large area of new collagen dyed dark blue was directionally aligned in the neonatal epithelial tissue, demonstrating an elevated amount of collagen deposition and improved tissue remodeling (Figure 6b), which attributed to the intrinsic collagen formation effect of NO.

M2 phenotype macrophages play a key role in inflammation regulation and wound remodeling by secreting growth factors, recruiting fibroblasts, and myofibroblasts transition. Diabetic wounds are usually accompanied by a deficiency of NO, which greatly weakens the inflammatory modulation and M2 macrophage distribution density. As shown in Figure 6c,d,g, extensive IL‐6 secretions and small quantity of M2 phenotype macrophages marked by CD163 were distributed in the control group and the HG‐MN group, indicating that a strong inflammatory response was experienced in these groups. Whereas, little amount of IL‐6 secretion and large counts of M2 phenotype macrophages were observed in the NHG‐MN+NIR group, indicating few signs of inflammatory response. These results were attributed to the positive effect of NO in modulating inflammatory response, thereby contributing to the rapid healing of diabetic wounds. The neovascularization degree is an important healing indicator that can be evaluated by the immunofluorescent stain of CD31 (endothelial cell markers) and *α*‐SMA (fibroblast markers). As revealed in Figure 6e, the new blood vessels distribution was sparse in the control group and HG‐MN group, indicating a low degree of vascularization. However, the blood vessel density in groups releasing NO molecule improved significantly, especially in the NHG‐MN+NIR group, reaching 2.9 ± 0.4% (Figure 6h). As an endogenous signal molecule, NO molecule possessed the ability to promote the migration and proliferation of endothelial cells, especially in hyperglycemic diabetic wounds, whose role in promoting wound healing can be fully revealed. These results strongly suggested that the NHG‐MN+NIR group would obtain superb progress in diabetic wound closure, as well as broad application value in the future.

## 3 Conclusion

In this paper, we have proposed and demonstrated a novel porous MOF MN patch enabling photothermal‐responsive NO delivery for diabetic wound healing. Due to the favorable photothermal response of the GO shell, the NHGs were imparted with the feature of temperature modulating under NIR, thereby facilitating the controlled release of NO molecules. When the NHGs were encapsulated in a porous PEGDA‐MN by template replication and UV polymerization method, the porous structure, larger specific surface area, and high mechanical strength of the integrated MN could promote the transdermal delivery of NO molecules more accurately and deeply. Owing to the excellent reparative capacity of NO molecules, the process of wound healing was obviously accelerated by NIR‐triggered NO release. It has been demonstrated that the resultant porous MOF MN patch integrated with photothermal responsive GO can promote vascularization, tissue regeneration, and collagen deposition in the diabetic wound bed. Thus, such photothermal responsive NO‐delivering MOF MN patch is expected for great application prospect in the area of wound healing.

## 4 Experimental Section

### Materials, Cell Lines, and Animals

PEGDA (average Mn 700), and 2‐hydroxy‐2‐methylpropiphenone (HMPP) were obtained from Sigma‐Aldrich. Cu(NO_3_)_2_ and BTC were purchased from Macklin Biochemical Technology Co., Ltd. GO was bought from XFNANO Material Tech Co., Ltd. The NIH/3T3 cells lines were obtained by the Cell Bank of the Chinese Academy of Sciences, Shanghai, China. Cells were conserved in DMEM (Gibco, USA). The MTT reagent was bought from Thermo Fisher Scientific. Antibodies of IL‐6, CD163, CD31, and *α*‐SMA were obtained from Servicebio Technology Co., Ltd. The male Sprague‐Dawley rats (150–200 g in weight) were obtained from Beijing Vital River Laboratory Animal Technology Co., Ltd. “Guidelines for the Care and Use of Laboratory Animals in China” was used as the basis for the treatment of laboratory animals. Animal Investigation Ethics Committee of Jinling Hospital has examined and approved all animal handling and experimental programs.

### Characterization

The brightfield images were shot and recorded by a microscope (Olympus, SZX16). The microstructures of MN patch and HKUST‐1‐derived microparticles were photographed by scanning electron microscope (SEM, Hitachi SU8010). The element composition of microparticles was conducted by energy‐dispersive spectrum (EDS, FEI Talos F200S). The characteristic peaks of HKUST‐1‐derived microparticles were collected by Fourier Transform Infrared Spectroscopy (FTIR, Tensor II). The diffraction peak of microparticles was tested by X‐ray diffractometer (XRD, Ultima IV). The thermal stability analysis was conducted by a thermogravimetric analyzer (TGA, 3Flex). The thermal images were recorded by a thermal imager (FLIR, E5xt). The nitrogen adsorption test of HKUST‐1‐derived microparticles was tested by an accelerated surface area porosimetry system (Micromeritics, 3Flex).

### Synthesis of the HKUST‐1‐Derived Microparticles

A conventional solvothermal method was conducted to prepare HKUST‐1‐derived microparticles. In short, Cu(NO_3_)_2_ (2.25 mmol) was dissolved in deionized (DI) water (7.5 mL) and mixed with ethanol (7.5 mL) containing BTC (1.25 mmol). The mixture was then ultrasonically treated for 15 min to fully blend. Afterward, GO (3 mg) was added to the compound to be mixed for 0.5 h, followed by a hydrothermal reaction in a Teflon‐lined container for 24 h at 120 °C. After that, the blending was rinsed with ethanol 3 times and dried in an 80 °C vacuum oven for 10 h to obtain the HGs. To modify the 4‐MAP onto HGs, the prepared HGs (40 mg) was transferred to a polytetrafluoroethylene‐lined solvothermal reactor preloaded with 4‐MAP (14.16 mg). Afterward, the reactor was heated at 140 °C for 12 h, and then washed with ethanol 3 times, drying in a 60 °C vacuum oven for 12 h. Thus, the 4‐MAP modified HGs was acquired.

### NO Loading of 4‐MAP Modified HGs

To bind the NO molecular with the internal secondary amino group to form NONOates groups, the 4‐MAP modified HGs were firstly placed in a 140 °C vacuum drying oven for 12 h. After that, it was placed in a pressure‐resistant glass bottle preloaded with pure NO (2 atm) for 1 h. Finally, 4‐MAP modified HGs loaded with NO molecular (NHGs) was obtained and stored in a glass bottle with nitrogen (N_2_) protection.

### Fabrication of Porous MOF MN Patch

The NHGs encapsulated porous MN patch (NHG‐MN) was prepared via the template replication method. First, PEGDA (60% v/v), acetic acid (5% v/v), HMPP (1% v/v), and NHGs were mixed and put in an ultrasonic cleaner for 5 min. Then DI water (35% v/v) and ammonium bicarbonate (6% w/v) were added to react completely for 10 min. After that, the pre‐gel solution was poured into the MN patch template and vacuumized for 1 min, and then the NHGs were precipitated in the needle tips by low‐speed centrifugation (100 rpm, 30 s). Finally, the pre‐gel solution in the template was solidified under UV light (365 nm, 100 W) for 20 s to obtain the expected HG‐MN and NHG‐MN. To prevent accidental decomposition of the NONOates group, the resultant MN arrays were conserved in nitrogen (N_2_) filled vials.

### Mechanical Strength Test

The MN patches were put on a horizontal surface of an electronic tension tester (Instron 5944) with their needle tips facing upwards. The compression force was recorded as long as the mechanical sensor touched the MN. Once reaching the maximum measured value of 100 N, the movement would stop. By conversion the linear part of the force‐displacement curve, Young's modulus can be obtained as the stiffness index of the MN patches.

### Controlled NO Release

The MN patches were conserved in a dry and airtight container protected with N_2_ in advance to eliminate the interference of air. Then, the MN patches were divided into four groups: PMN, HG‐MN, NHG‐MN, and NHG‐MN+NIR group (irradiate 10 min every half an hour). First, the N_2_ was endowed with a relative humidity of 85% through a container containing potassium chloride saturated salt solution. The humidified N_2_ was pushed into the MN patch chamber, and the NO released from NHG‐MN was bubbled into a sealed vial containing 3 mL DI water at room temperature. In the vial, NO reacted with water to form nitrite ions, and the resultant nitrite solution was collected at predetermined intervals. Griess assay was utilized to detect the nitrite concentration, which could indirectly reflect the releasing amount of NO. The standard curve was established using the nitrate standard sample. In detail, nitrite solution sample (100 µL) was mixed with sulfonamide solution (1% w/v sulfonamide in 5% v/v phosphoric acid, 100 µL), shaking the mixed solution for 10 min. Then, *N*‐(1‐naphthyl)‐ethylenediamine dihydrochloride solution (NED, 0.1% v/v, 100 µL) was added and incubated for 10 min. Finally, a microplate reader (BioTek EPOCH 2) was used to measure the OD value at 540 nm.

### Copper Ion Release Experiment

The release of Cu^2+^ required water as medium to be decomposed from HKUST‐1. Thus, Cu^2+^ concentration was determined by BCO spectrophotometric method. Firstly, MN patches in different groups (PMN, HG‐MN, NHG‐MN, and NHG‐MN+NIR group) were cut into a square of 1 cm^2^. After that, the samples were immersed into PBS solution (2 mL) to simulate the physiological environment in vivo. In this process, the filtrate was collected at regular intervals and the absorbance at 600 nm was determined by using BCO spectrophotometric method. Finally, Cu^2+^ concentration was calculated with the Cu^2+^ standard curve.

### Cytotoxicity Test

The cytotoxicity of HG‐MN, NHG‐MN, and NHG‐MN+NIR groups was determined by the MTT assay. First, the MN patches were washed in sterile PBS to remove impurities from the surface. Then, the MN patches in different groups were immersed in a serum‐containing NIH/3T3 cell culture medium, placing in a 37 °C incubator for 24 h. Cells with an initial concentration of 2 × 10^3^/well were incubated in a 96‐well plate overnight to adhere cells to the lower wall. Finally, the original cell medium was replaced with filtrate (100 µL) from the experimental groups. For the first 3 days, a microplate reader (BioTek EPOCH 2) was used to detect the absorbance at 490 nm treated by the MTT assay.

### Establishment of Diabetic Rat Model

Streptozotocin (STZ, 1% w/v) was dissolved in the citric acid buffer, followed by the injection at a dose of 60 mg kg^−1^ into the abdominal cavity to build a type I diabetic rat model. The blood glucose was measured and recorded by a blood glucometer 3 days later.

### Wound Healing Experiment In Vivo

The type I diabetic rats were divided into five groups at random. The first group was the control group. The second group was the PMN group, in which the applied MN patches only contained PEGDA without any other active components. The third group was the HG‐MN group without NO molecules loaded inside them. The fourth group was the NHG‐MN group without NIR treatment. The fifth group was the NHG‐MN+NIR group. The NIR irradiation was performed once a day, 5 min each time. After abdominal anesthetic, the hair on the rats’ back was shaved off, and then four parallel wounds with a diameter of 1 cm were cut out on the dorsal skin by a scalpel. Experimental rats were kept in cages with adequate food and water in clean animal room. The wounds recovery condition was recorded on the first 13 days. On the 13th day, the rats were euthanized, with their healed tissues excised for further examination.

### Histological Analysis

Paraformaldehyde (Beyotime, 4% (w/v)) was used to fix the healed tissue samples immediately after removal from rat back. The samples were then progressively dehydrated and encased in paraffin (Huayong, China). Finally, a slicer (Leica, RM2265) was used to slice the samples to a thickness of 5 µm for subsequent examination (H&E staining, Masson trichrome staining, immunohistochemical, and vascularization analysis).

### Statistical Analysis

Data analysis was conducted using GraphPad Prism 8 software (GraphPad Software, San Diego, CA, USA). Data were presented in the form of mean ± standard deviation (SD). Statistical differences were determined using one‐way analysis of variance (ANOVA). The levels of significance were labeled with *p < 0.05, **p < 0.01, ***p < 0.001.

## Conflict of Interest

The authors declare no conflict of interest.

## Author Contributions

S.Y. and Y.T.W. contributed equally to this work. Y.J.Z., Y.L., and Y.A.W. conceived the idea and designed the experiment; S.Y. and Y.T.W. conducted experiments and data analysis; Y.T.W., J.J.C., Y.R.Y., Y.J.Z., Y.L., and Y.A.W. wrote the manuscript.

## Supporting information

Supporting Information

## Data Availability

Research data are not shared.
