# Supplementary material for: Porous MOF Microneedle Array Patch with Photothermal Responsive Nitric Oxide Delivery for Wound Healing
Source: Adv Sci (Weinh). 2021 Nov 16;9(3):2103449. doi: 10.1002/advs.202103449 (PMC8787387; doi:10.1002/advs.202103449)
Supplement: Supplementary file 1 — Supporting Information [file ADVS-9-2103449-s001.pdf]

ADVANCED SCIENCE
Open Access

Supporting Information

for *Adv. Sci.*, DOI: 10.1002/advs.202103449

Porous MOF Microneedle Array Patch with Photothermal Responsive Nitric Oxide Delivery for Wound Healing

Shun Yao, Yuetong Wang, Junjie Chi, Yunru Yu, Yuanjin Zhao, Yuan Luo*, Yongan Wang**

Supporting Information

Porous MOF Microneedle Array Patch with Photothermal Responsive Nitric Oxide Delivery for Wound Healing

Shun Yao, Yuetong Wang, Junjie Chi, Yunru Yu, Yuanjin Zhao, Yuan Luo*, Yongan Wang**

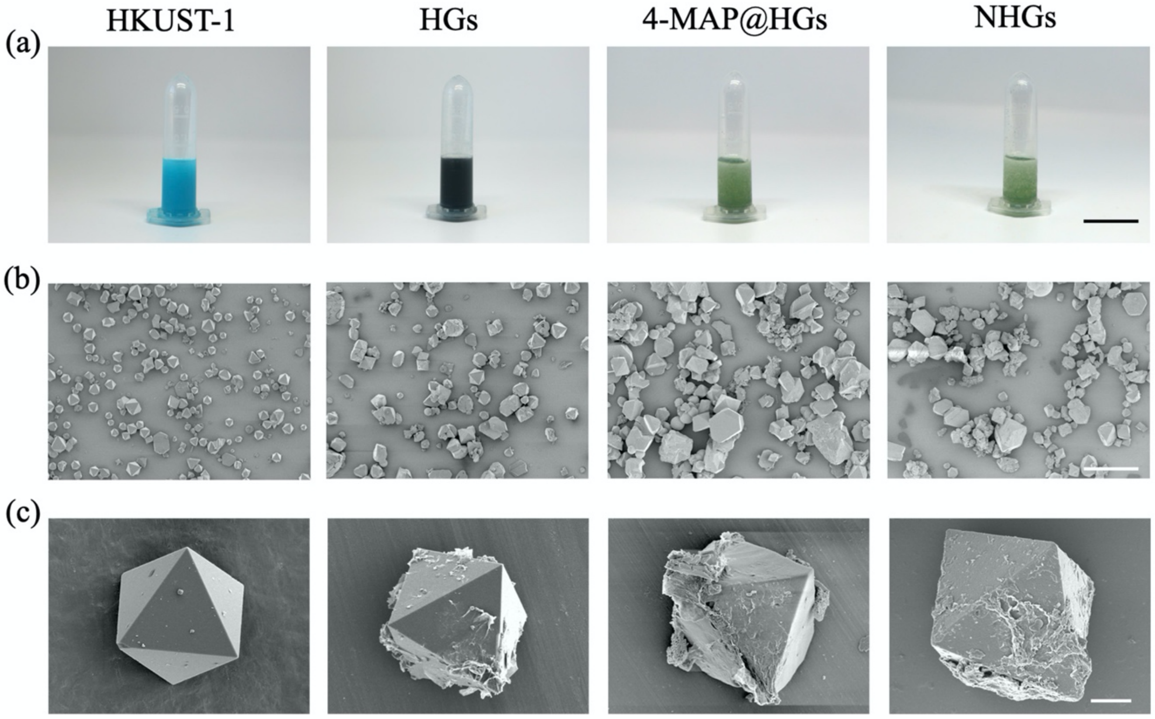

Figure S1. Characterization of NHGs synthesis process: (a) Color change of the HKUST-1-derived microparticles dispersion; (b) SEM images reflecting the aggregation of HKUST-1-derived microparticles; (c) SEM images of the representative individual microparticle. Scale bars are 1 cm in (a), 100 µm in (b), and 5 µm in (c).

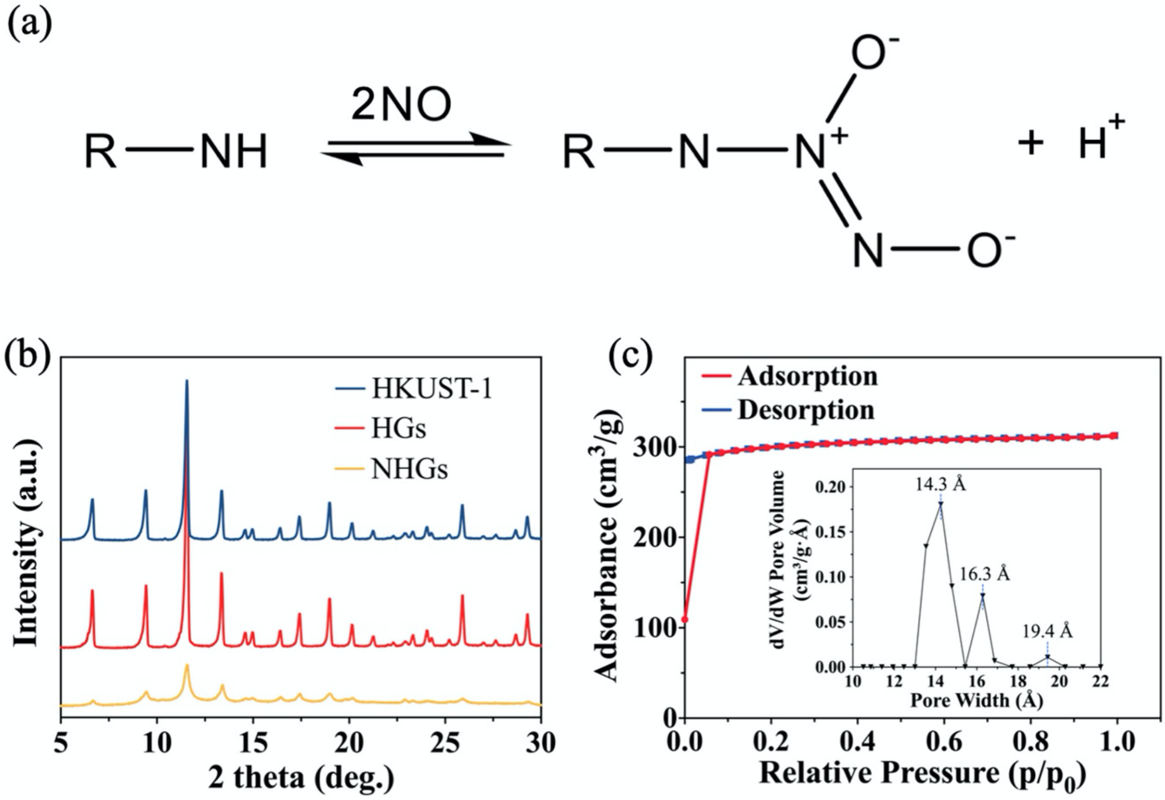

Figure S2. (a) Reversible NONOates formation on secondary amino group; (b) XRD spectrum of the HKUST-1-derived microparticles; (c) The nitrogen absorption curve and porosity of HKUST-1 microparticles.

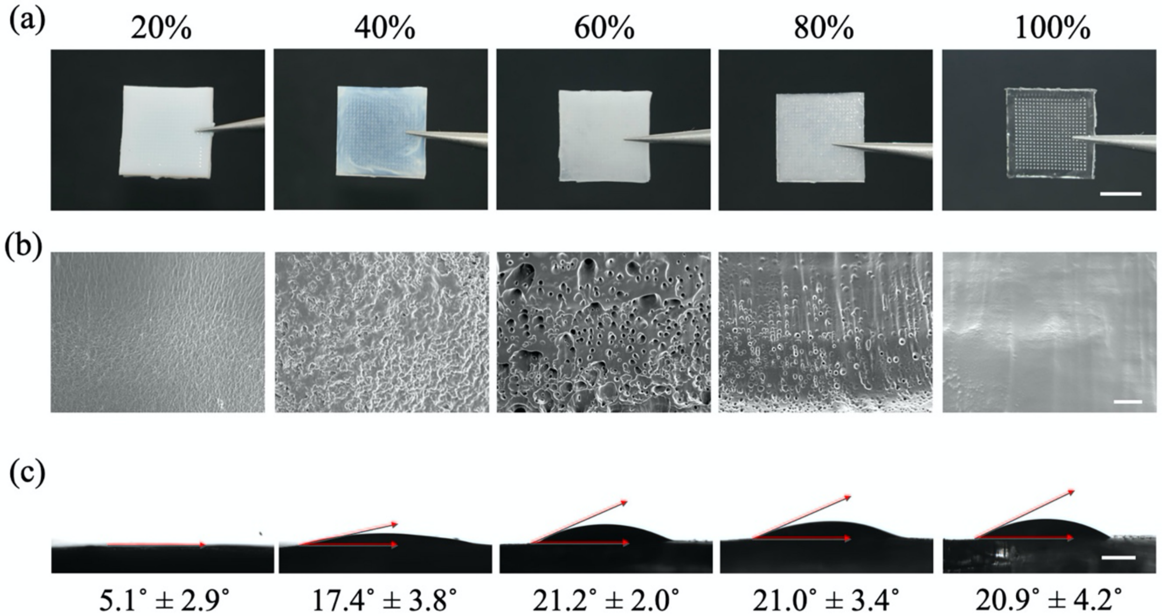

Figure S3. (a) Optical images of PEGDA-MN with different PEGDA concentrations; (b) SEM images of the porous structure on the MN needle-tips; (c) The hydrophilicity test of different MN surfaces. Scale bars are 0.5 cm in (a), 10 μm in (b), 1 mm in (c).

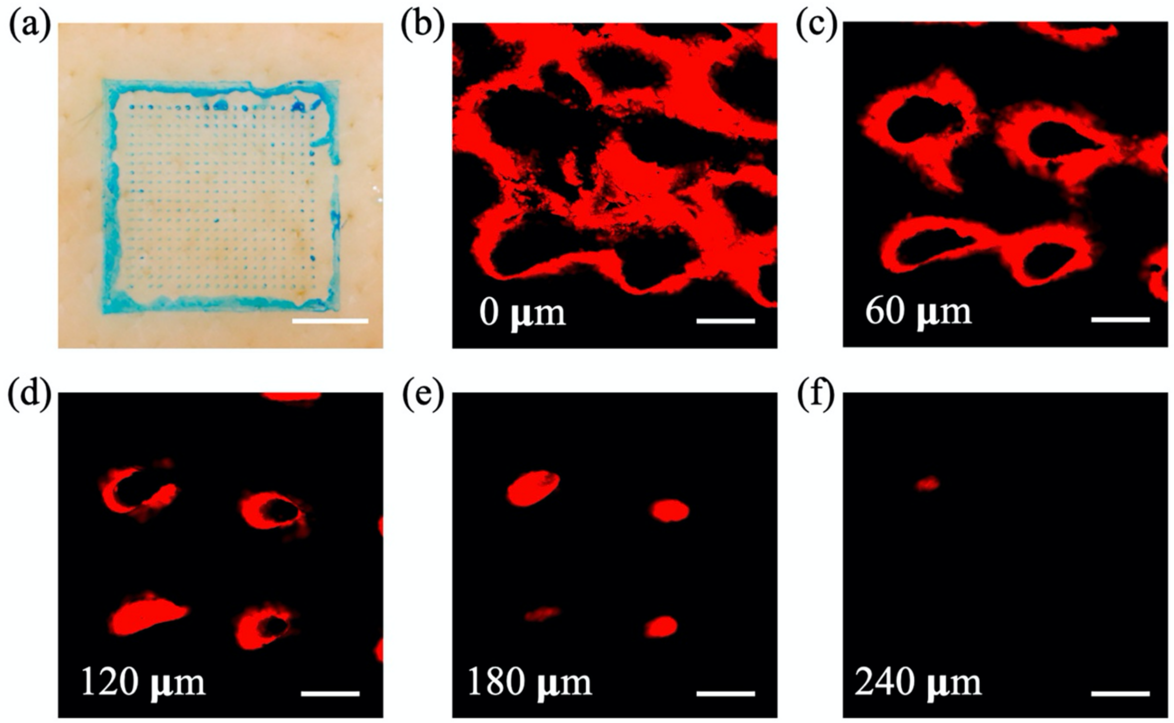

Figure S4. (a) The grooves left after removing the applied PEGDA-MN stained with methylene blue; (b-f) The LSCM images showing the penetrating depth of needle tips in the simulated skin. Scale bars are 500 μm in (a) and 200 μm in (b-f).

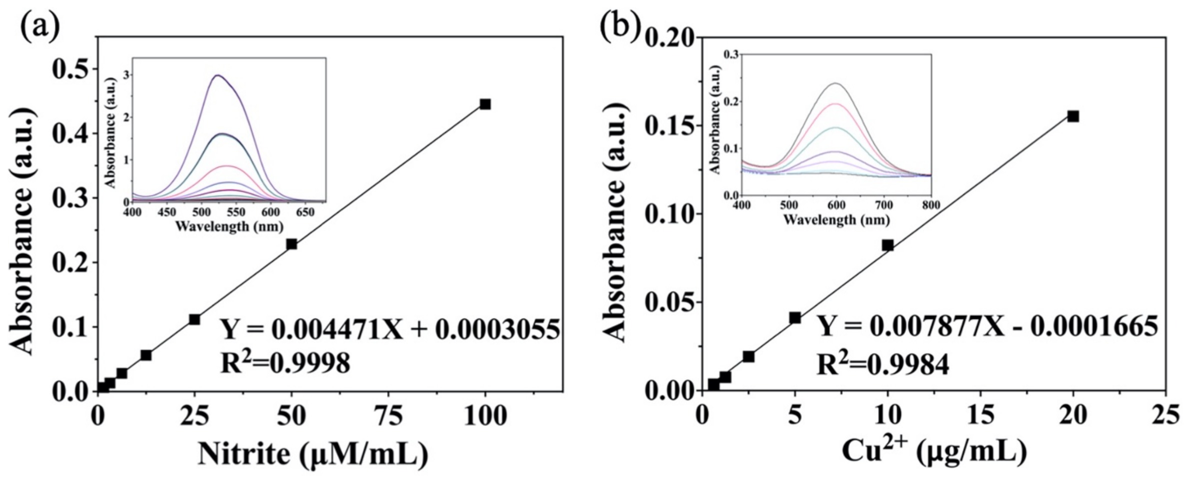

Figure S5. (a) Nitrite standard curve measured by the Griess assay; (b) Cu^{2+} standard curve measured by the BCO spectrophotometric method.

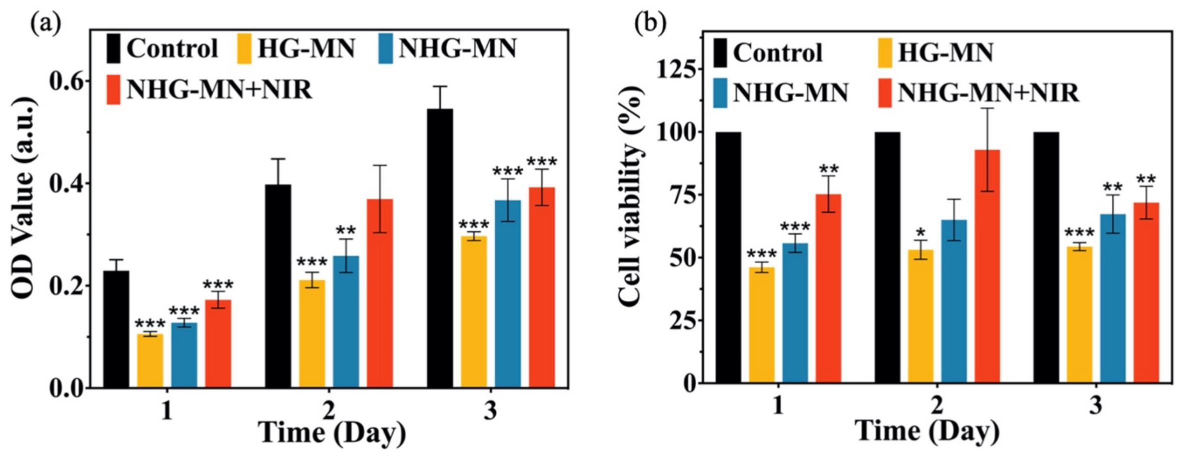

Figure S6. Biocompatibility of porous MOF MN patch: (a) The OD value of absorbance at 490 nm and (b) the corresponding cell viability. Data are shown as mean ± SD (n = 3) and analyzed using the one-way ANOVA test. Significances are presented by *p < 0.05, **p < 0.01, ***p < 0.001.

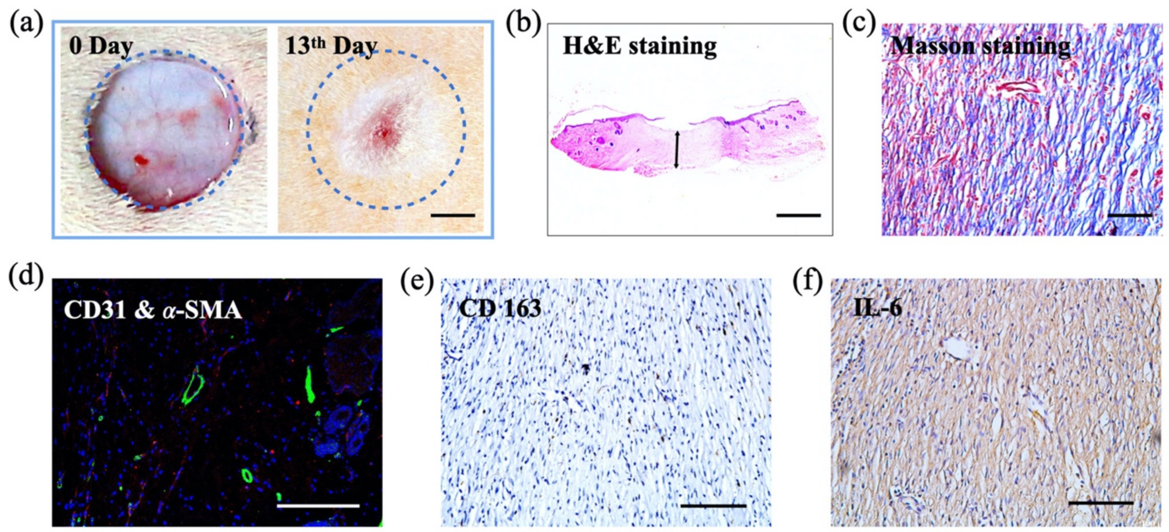

Figure S7. Wounds treated by PMN: (a) Typical images of the wound on day 0, and day 13; (b) H&E staining of the wound tissue on day 13; (c) Masson trichrome staining of the wound tissue on day 13; (d) Vascularization analysis of wound tissue stained by CD31 and α-SMA on day 13; (e) Immunohistochemistry staining of CD163 on day 13; (f) Immunohistochemistry staining of IL-6 on day 13. Scale bars are 3 mm in (a), 2 mm in (b), and 100 μm in (c-f).
